# The Contribution of Chemoattractant GPCRs, Formylpeptide Receptors, to Inflammation and Cancer

**DOI:** 10.3389/fendo.2020.00017

**Published:** 2020-01-24

**Authors:** Weiwei Liang, Keqiang Chen, Wanghua Gong, Teizo Yoshimura, Yingying Le, Ying Wang, Ji Ming Wang

**Affiliations:** ^1^Department of Immunology, School of Basic Medical Sciences, NHC Key Laboratory of Medical Immunology, Peking University, Beijing, China; ^2^Cancer and Inflammation Program, Center for Cancer Research, National Cancer Institute at Frederick, Frederick, MD, United States; ^3^Basic Research Program, Leidos Biomedical Research, Inc., Frederick, MD, United States; ^4^Department of Pathology and Experimental Medicine, Graduate School of Medicine, Dentistry and Pharmaceutical Sciences, Okayama University, Okayama, Japan; ^5^CAS Key Laboratory of Nutrition, Metabolism and Food Safety, Shanghai Institute of Nutrition and Health, Chinese Academy of Sciences, Shanghai, China

**Keywords:** FPRs, regulation, chemotaxis, inflammation, infection, immunity, cancer

## Abstract

A hallmark of inflammatory responses is leukocyte mobilization, which is mediated by pathogen and host released chemotactic factors that activate Gi-protein-coupled seven-transmembrane receptors (GPCRs) on host cell surface. Formylpeptide receptors (FPRs, Fprs in mice) are members of the chemoattractant GPCR family, shown to be critical in myeloid cell trafficking during infection, inflammation, immune responses, and cancer progression. Accumulating evidence demonstrates that both human FPRs and murine Fprs are involved in a number of patho-physiological processes because of their expression on a wide variety of cell types in addition to myeloid cells. The unique capacity of FPRs (Fprs) to interact with numerous structurally unrelated chemotactic ligands enables these receptors to participate in orchestrated disease initiation, progression, and resolution. One murine Fpr member, Fpr2, and its endogenous agonist peptide, Cathelicidin-related antimicrobial peptide (CRAMP), have been demonstrated as key mediators of colon mucosal homeostasis and protection from inflammation and associated tumorigenesis. Recent availability of genetically engineered mouse models greatly expanded the understanding of the role of FPRs (Fprs) in pathophysiology that places these molecules in the list of potential targets for therapeutic intervention of diseases.

## Introduction

**Figure 1:**
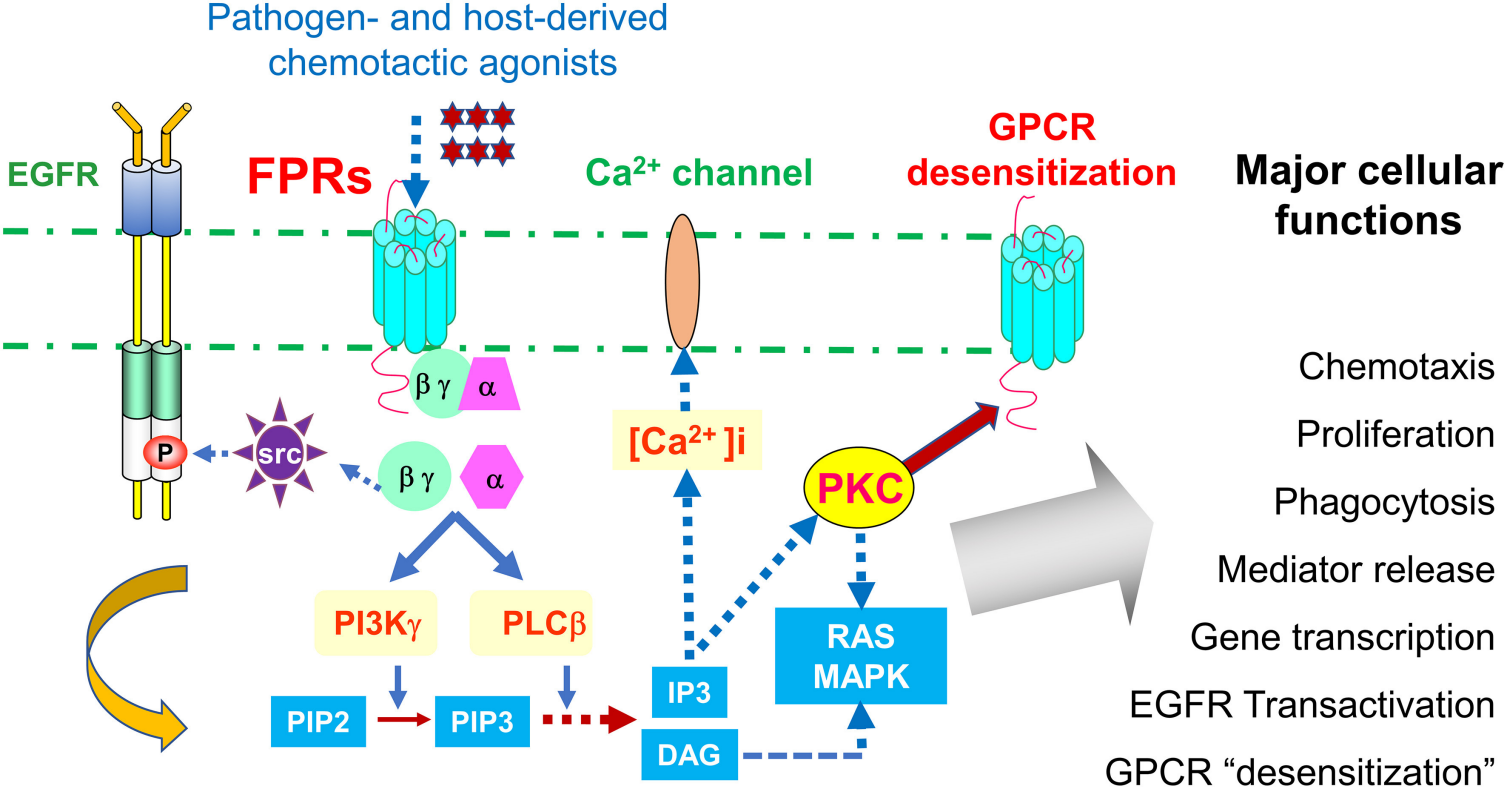
Signaling pathways of FPRs. FPRs (Fprs in mice) sense bacteria chemotactic PAMPs and host tissue-derived chemotactic DAMPs, and mediate Gi-protein-associated signaling cascade including calcium mobilization and activation of protein kinases culminating in cell migration, proliferation, phagocytosis, and gene transcription. Activation of FPRs in epithelial and cancer cells also transactivate EGF receptor, which cooperate with FPRs in tumor cell activation. In addition, FPRs possess the ability to heterologously “desensitize” other chemoattractant GPCRs, notably chemokine GPCRs involved in HIV-1 fusion and neutrophils recruitment in trauma and infection. Therefore, desensitization of HIV-1 co-receptors by FPRs may offer novel approach to therapeutic drug design, desensitization of neutrophils recruiting chemoattractant GPCRs by FPR1 is detrimental to host defense in trauma and secondary infection.

Leukocyte infiltrate the site of inflammation, immune responses and cancer by sensing chemotactic signals that form a gradient inside and at the vicinity of tissue microenvironment. This cell infiltration is mediated by a large family of chemoattractant receptors with seven-transmembrane, Gi-protein coupled features (GPCRs), which include “classical chemoattractant GPCRs” and “chemokine GPCRs.” Formylpeptide receptors (FPRs in human, Fprs in mice) belong to the “classical chemoattractant GPCRs” (1), initially cloned from neutrophils, but have been identified in many cell types including immune cells and cells of the non-hematopoietic origin (2–5). Humans have three FPRs: FPR1, FPR2, and FPR3, which share about 70% identity at the amino acid level. FPR1 was the first named member of this receptor family for bacterial formylated peptides, such as formyl-methionyl-leucyl-phenylalanine (fMLF) (6). Both FPR1 and FPR2 are highly expressed in many tissues and organs with cells of the hematopoietic and non-hematopoietic origin (7). Human FPR3 is more specifically expressed by monocytes, and probably also dendritic cells (DCs), with only one better known host-derived, endogenous peptide agonist F2L (8, 9), presumably participating in DC recruitment *in vivo*. It is well-established that stimulation of FPRs (Fprs) by agonists triggers the dissociation of trimeric G-proteins coupled to intracellular domains of the receptors resulting in activation of a signaling cascade that initiates multiple cell functions (Figure 1).

The major function of FPRs is thought to mediate cell chemotaxis in response to agonists, but when activated, they also enhance phagocytosis of death tissues and bacteria by neutrophils (10, 11), mediator (such as ROS) generation, NET formation, cytokine release, and increased phagocytosis. These receptors additionally promotes wound healing and gut mucosal development (12, 13). In fact, FPR1 and FPR2 play critical roles in the process of multiple diseases. For instance, FPR2 may promote the malignancy of human colon cancer, while FPR1 is linked to the progression of human glioblastoma multiforme (GBM) (14, 15). One rather interesting property of FPR1 was to inhibit the progression of gastric cancer as a potential “tumor suppressor” (16). With these seemingly “opposing functions” in cancer progression, further studies of this aspect of FPRs should benefit the development of new anti-cancer strategies.

While abnormal expression of FPRs can be harmful, active FPRs was essential for host defense against the invasion and expansion of pathogenic bacteria, including *Candida albicans* that forms biofilms in the gut and *Vibrio harveyi* that may aggressively infiltrate the protective layer of the colon mucosal surface (17). Studies have shown that Fpr2 confers protection against sepsis-mediated tissue damage in mice and both Fpr1 and Fpr2 are indispensable for mouse resistance to *Listeria* infection (18–20). A rather surprising function for FPRs was their involvement in anxiety-linked disorders and abnormal animal behavior, with as yet to be elucidated mechanisms (21). Further, FPRs may act as “mechano-receptors (or sensors)” on large arteries to maintain proper plasticity vital to the normal cardiovascular function (22).

Although FPRs (Fprs) are in general believed to transmit “proinflammatory signals” in leukocytes, they are also reported to mediate “anti-inflammatory” or “resolving” signaling in some cell types (23). The latter was mainly attributed to the findings with an endogenous FPR1/FPR2 ligand Annexin A1 (Anxa1) and its peptide fragments. The molecular basis for the capacity of these ligands to elicit a divergent signaling cascade was attributed to their tentative binding to different domains of the receptors (24). FPRs have also been reported to form homo- or heterodimers in the presence or absence of ligand binding. But this remains a controversial issue, with unclear pathophysiological implications (25, 26).

Another important pathophysiological characteristic of FPRs (Fprs) is their capacity of homologous desensitization in which activation of the receptor by a ligand causes the unresponsiveness of the receptor to subsequent stimulation by the ligands. Interestingly, FPRs (Fprs) also demonstrate a unique capability to result in heterologous desensitization of other chemoattractant GPCRs. More importantly, FPRs appear to be on top of a desensitization hierarchy, where FPRs seem to possess a “one directional desensitization” capacity vs. other chemoattractant GPCRs, especially chemokine GPCRs, through a protein kinase C mediated signaling pathway (27). Desensitization of chemokine receptors CCR5 and CXCR4 on immune cells by FPR ligands resulted in the loss of the capacity of these GPCRs to act as HIV fusion co-receptors therefore suggest a unique opportunity for development of novel anti-HIV therapeutic agents (28, 29).

The pathophysiological significance of receptor desensitization was demonstrated in a recent study in which mitochondrial and bacterial formylated peptides were shown to activate FPR1 on neutrophils to desensitize cell response to chemokines and leukotrienes derived at the sites of trauma or infection, which is detrimental to host defense. Blockade of FPR1 by using receptor inhibitors (such as cyclosporin H) or gene deletion (*Fpr1 KO*) preserved normal neutrophil bacterial phagocytosis or superoxide production in response to trauma or infection. Therefore, mitigating the “desensitizing” activity of FPR1 (Fpr1) has shown profound effect on protecting the host from systemic sterile inflammation and secondary infection following tissue injury or primary infection (30).

The diverse functions of FPRs (Fprs) are attributable to their interaction with a plethora of pathogen-associated and damage-associated chemotactic molecular patterns (PAMPs and DAMPs) (23, 31–34). However, due to the mounting evidence for the important functions of FPRs (Fprs) in patho-physiological conditions, this review would only cover relatively narrow aspects of these receptors, i.e., their role in inflammatory responses and cancer progression. The readers are referred to additional reviews and original articles for more details about topics of special interest in FPR (Fpr) research (23, 31–33, 35).

## FPRs in Inflammation

Based on their capacity to recognize a variety of chemotactic PAMPs and DAMPs, the primary function of FPRs (Fprs) are historically attributed to the host anti-microbial defense and inflammatory responses.

### The Essential Role of FPRs in Host Defense Against Bacterial Infection

*Listeria monocytogen* is an opportunistic pathogen that mainly infects immunocompromised human subjects with high lethality (30%) (33, 36). The major mechanisms for host resistance to *Listeria* dependent on rapid neutrophil recruitment to the infected tissues and organs. Although neutrophils express several chemoattractant GPCRs, Fprs were found to mediate the early wave of cell infiltration in response to bacteria-derived chemotactic PAMPs.

The first evidence for the importance of Fpr1 in *Listeria* infection was provided in an early study which demonstrated increased susceptibility of *Fpr1* KO mice to infection (18, 20). With the availability of additional genetically engineered mice, evaluating the contribution of each or both Fprs to host defense was made feasible. When *Listeria* was injected i.v. into wild type (WT) mice, high level neutrophil accumulation occurred in the liver in <30 min with maximal cell infiltration at 4 h, when neutrophil-specific chemokines CXCL1 and 2 that activate the GPCR, CXCR2 (37), were only detectable at low levels. While in Fpr1 or Frp2 single KO mice, early recruitment of neutrophils into the infected liver is significantly diminished, which was almost absent in the liver of infected Fpr1 and Fpr2 double KO mice (20). The involvement of Fprs in mouse resistance to *Listeria* infection was further indicated by the ability of *Listeria* to produce chemotactic agonists for both Fpr1 and Fpr2 (38) and while mice deficient in a single Fpr showed increased bacterial load in the liver, deficient in both Fpr1 and Fpr2 resulted in greater bacterial load in the liver and animal mortality after infection. These findings modified an existing paradigm in which the pattern recognition receptor TLR2 on host cells was activated by bacterial lipoprotein to elicit the production of CXCR2 chemokines that initiate neutrophil accumulation at the infected sites. The novel observations prompted the modification of the paradigm by clear evidence that Fprs antecede CXCR2 in rapidly mobilizing the first wave of neutrophil recruitment by sensing *Listeria* chemotactic signals.

Based on the ability of FPRs (Fprs) to interact with a wide range of pathogen-derived chemotactic PAMPs, it is conceivable that these GPCRs also promote neutrophil accumulation and host resistance in infection models of other bacteria (33), which include *Streptococcus pneumonia* causing meningitis (39). Also, in chemically induced colitis in mice deficient in Toll-interacting-protein (Tollip), neutrophil infiltration in gut lesions is reduced due to lower level expression of Fpr2 (40). In addition, mouse neutrophils expresses reduced levels of FPR2 (Fpr2) in a sepsis model (19, 41), thus failing to infiltrate the site of infection. It is interesting that neutrophils in such sepsis subjects may be in an “inflamed” but “incompetent” state causing higher morbidity and mortality of the host (42).

A recent study demonstrated an important role of Fpr2 in orchestrating the protection of colon mucosa from infection by *Citrobacter (C) rodentium*, which is an attaching and “effacing” intestinal mouse pathogen that shares similar virulent patterns with human enteropathogenic *E. coli*. The expression of Fpr 2 on the surface of colon epithelial cells was upregulated during *C. rodentium* infection. Although both WT and *Fpr2 KO* mice are infected by *C. rodentium, Fpr2 KO* mice displayed a significantly slower recovery due to reduced rate of bacteria clearance. The active involvement of Fpr2 in host defense against *C. rodentium* infection was further shown by increased susceptibility of *Fpr2 KO* mice to lower dose bacterial inoculation with a 100% colonization vs. 30% in WT mice. There was also a more severe colitis in *Fpr2 KO* mice after bacterial infection with increased bacteria in contact with colon epithelial cells. In addition, *Fpr2 KO* mice showed a higher *C. rodentium* load in the spleen after infection and there was an enhanced translocation of *C. rodentium* and *E. coli* across an artificial mucosal surface to the basolateral compartment established *in vitro*, in the absence of functional Fpr2. Furthermore, compared to WT mice, the colon mucosa of *Fpr2 KO* mice lacked striated inner mucus layer with decreased production of mucus. These findings indicate that Fpr2 protects the colon against infection by supporting a normal mucus barrier (43). Therefore, FPRs (Fprs) stand in the forefront of anti-microbial host defense.

### Essential Participation in Chemotaxis Signal Replay in Wounds by FPRs

Neutrophils are major inflammatory cell infiltrates that rapidly accumulate at the site of acute skin wounds (44). This process is initially thought to be controlled by a number of chemoattractants such as CXCL8, CXCL10 (44–46) and CXCL1, chemokines that activate the GPCR, CXCR2, on neutrophils. But in an acute skin-wound model, mouse Fpr1/Fpr2 are found to be first responders on neutrophils to chemotactic ligands released at the site of injury (13), followed by a sequential participation of other GPCRs resulting in subsequent waves of cell infiltration in association with phagocytosis, release of superoxide and eventual closure of the wound. In Fpr1 and 2 double deficient mice, the healing process of wounded skin is significantly delayed associated with reduced accumulation of neutrophils. Thus, both Fpr1/Fpr2 are critically involved in the normal healing process of skin wound as the first sensors of tissue-derived chemotactic DAMPs (13). As supporting evidence, in a sterile ear skin wound model in mice, several chemoattractant GPCRs including leukotriene B4 receptor, Fpr2, and CXCR2, cooperate to orchestrate a dynamic neutrophil “swarming” inside the wound and its immediate surrounding area (47). Therefore, concerted participation of chemoattractant GPCRs contributes to the host responses vital to the normal healing of acute skin wound.

### Cooperation of Fpr2 With Other Chemokine GPCR in Mediating DC Trafficking *in vivo*

Asthma, as a chronic airway inflammation and hyperresponsiveness, is mainly a Th2 immune response, with increased production of IL-4, IL-5, and IL-13 and serum IgE (13, 48). The expression of an Fpr2 ligand, CRAMP, is also enhanced in the allergic lung (49). The most effective remedy for asthma thus far remains to be glucocorticoids with other, yet limited, drugs such as bronchoalveolar dilatators and sputum thinners, to ameliorate complications. However, prolonged use of these therapies may compromise antibacterial defense regarding the lung and epithelial cell function, as a result of reduced production of anti-microbial molecules such as CRAMP in the airway. CRAMP and the human counterpart peptide, LL-37, activate Fpr2 (and FPR2) to induce myeloid cell chemotaxis, thus presumably contributing to the recruitment of such cells in the diseased lung (50–53).

DCs are the effective antigen-presenting cells and play an important role the pathogenesis of allergic airway inflammation (54). Upon challenge by allergens, such as virus or endotoxin, TLR agonist PAMPs or environmental stimulants induce chemokine CCL2 that rapidly recruits CD11b^+^ DC precursors through a GPCR, CCR2, into small airways where these CD11b^+^ DC precursors differentiate into inflammatory CD11c^+^DCs during disease progression. When the cells become mature, they migrate into draining lymph nodes to prime T cell responses (55). It is therefore predictable that in an OVA-induced asthma model, CCR2 deficiency (CCR2 KO) results in defective trafficking of CD11c^+^DCs loaded with antigens during maturation in the lung tissue, leading to weakened Th2 immunity (55–58). However, this mechanistic basis is recently substantiated by including Fpr2 in the key phases of allergic airway inflammation (54). In fact, there is a tightly orchestrated DC trafficking in allergic airway inflammation, in which CCR2 mobilizes monocytic DC precursors from the bone marrow (BM) into the circulation (58, 59), where CCR2 continues to guide the cells into the perivascular regions of the inflamed lung where the precursors differentiate into immature DCs by contacting PAMPs and DAMPs in the lung (54). Meanwhile, the levels of CCR2 on immature DCs were reduced, but with increased expression of Fpr2, which mobilizes the cells into the peribronchiolar regions by sensing a chemotactic gradient of CRAMP (54). CRAMP also promotes the maturation of DCs in cooperation with TLR agonistic PAMPs and pro-inflammatory cytokines such as TNFα or LPS (60). A new expression pattern of chemoattractant GPCR on cell surface then ensured with down-regulation of Fpr2 but elevation of the DC homing GPCR, CCR7, that enables homing of matured DCs to draining lymph nodes. Therefore, a fine-tuned coordination of chemoattractant GPCRs on immature, then on mature, DCs, from CCR2, via Fpr2, with CCR7 as the final player of a relay of chemotaxis to complete the final segment in cell homing from the lung to draining lymph nodes.

## FPRs in Cancer

In addition to regulating immune cell trafficking and inflammatory responses, FPRs have been implicated in cancer progression.

### Regulation of M1 vs. M2 TAMs in Tumor by Fpr2

Macrophages are composed of M1 and M2 (or alternatively activated) types after differentiation in tissues under influence of environmental signals. M1 macrophages produce pro-inflammatory cytokines to enhance host resistance to pathogens and prime Th1 responses. In contrast, the M2 macrophages with a rather complex composition are (61, 62) more prone to phagocytosis and mediate parasite control, tissue remodeling, angiogenesis and tumor progression.

TAMs are a major component within the tumor stroma and mostly function as M2d subtype of macrophages (63, 64). At the initial stage of tumor formation, some infiltrating TAMs present as an IL-12^high^ IL-10^low^ M1 phenotype and may delay tumor growth. However, with the progression of tumors, TAMs often switch to an IL-12^low^/IL-10^high^ M2 phenotype with reduced tumoricidal capacity (63) but favoring tumor progression and metastasis (65, 66).

In a study of mouse Lewis lung cancer (LLC) model, Fpr2 KO mice subcutaneously implanted LLC cells suffered from more rapidly growing tumors with significantly shortened survival compared with WT counterparts. In contrast, in Fpr2 transgenic mice, subcutaneously implanted LLC tumors grew more slowly (67). Pathology studies of tumor tissues detected increased number of TAMs in tumors grown in Fpr2 KO mice and the macrophages isolated from Fpr2 KO mice showed a more-potent chemotactic response to LLC-derived supernatant that contained high concentrations of the chemokine CCL2. Thus, CCL2 is a major chemoattractant for TAM infiltration of LLC tumor. Furthermore, Fpr2 KO mouse macrophages expressed higher levels of a chemokine GPCR, CCR4 that recognizes CCL2 (68, 69). It is of interest that treatment of WT mouse macrophages with Fpr2 antagonists increased their chemotactic response to CCL2 mediated by elevated cell surface CCR4. In addition, LLC cell supernatant and Fpr2 ligands polarize WT macrophages to an M1 phenotype (67). Therefore, Fpr2 favors M1 polarization of macrophages to promote anti-LLC host defense. However, there is a caveat in generalizing the results derived from LLC transplantation model because not all tumors produce copious levels of CCL2 and the nature of LLC-derived Fpr2 agonists is not fully explained by the presence of one of the Fpr2 agonists CRAMP. This calls for further studies to elucidate the mechanisms underlying the capacity of Fpr2 to promote M1 polarization of macrophages that limit tumor growth.

### Protection of Host From Tumorigenesis by FPRs

In addition to myeloid cells, FPRs are expressed by many cells of the non-hematopoietic origin, including intestinal epithelial cells and have been shown to protect colon mucosal homeostasis.

Ulcerative colitis (UC) is a major causative factor of colorectal cancer (70). Chronic inflammation accompanying UC is linked to neoplastic transformation of the mucosal epithelia. The link between UC and cancer is attributed to submucosal inflammation in the colon initiated by contact with a skewed intestinal microbiome that promotes malignant transformation (71). It is postulated that the ability of intestinal epithelia to adapt to microbiome composition change is important for controlling inflammation but also for preventing tumor formation. In human, FPR1 is located along the lateral membrane of colonic crypt cells. Bacterial fMLF stimulates epithelial growth through FPR1 to restore the integrity of the colon mucosa (72). In mice, chemotactic Fpr agonists including Anx A1, fMLF, and viable *Lactobacillus rhamnosus* GG stimulate Fpr1 on colon epithelial cells to generate reactive oxygen species via enterocyte NADPH oxidase 1 (NOX1), which causes rapid phosphorylation of focal adhesion kinase (FAK) and extracellular signal-regulated kinase (ERK) (73). FPR1 on colon epithelial cells also promotes the motility and growth of enterocytes adjacent to colonic wounds (74). Colon crypts of Fpr1 deficient mice contain increased number of proliferating epithelial cells and that migrate more slowly along the crypt-villus axis, regardless an apparently normal tissue architecture. Thus, Fpr1 protects the homeostasis of the colon epithelia by promoting the restitution of the damaged mucosa (72).

Lysates of commensal bacteria as well as the bacterial product fMLF activate MAP Kinase signaling cascade in mouse colon in an FPR-dependent manner (15). In *Fpr* KO mice, Fpr2, but not Fpr1, is a major component in the maintenance of colon epithelial cell proliferation (12). In mouse colon, Fpr2 is expressed on the apical and lateral surface of crypt epithelial cells and fMLF stimulates epithelial cell renewal. Also, in *Fpr2* deficient mice, colon epithelial cells are defective in response to commensal bacterium-stimulated crypts development, with reduced severity in chemically induced colitis but lapsed recovery of mucosa from chronic damage in association with increased tumorigenesis. However, unlike Fpr2, despite the ability of Fpr1 to mediates fMLF-elicited migration and activation of colon epithelial cells, the length of colon crypts in *Fpr1* KO mice are normal (12). In addition, colons of mice deficient in both *Fpr1* and *Fpr2* showed shortened crypts that simulate the phenotype of *Fpr2* single deficient mice. These results confirm the capacity of Fpr2 to protect the colon by interaction with microbiome- and host-derived agonists.

### Dual Roles Shown by FPRs in Cancer Progression

As discussed earlier, certain opposing roles of FPRs have been reported in the progression of malignant tumors. It is now clear that under physiological circumstances, FPRs on normal cells are critical for anti-microbial responses and for controlling inflammation, immune responses and epithelial homeostasis. However, FPRs are also expressed by some malignant tumor cells and are activated by chemotactic PAMPs or endogenous DAMPs to their advantage. This is shown by studies with human gastric cancer (GC) cells with aberrantly expressed FPRs, which mediate epithelial-mesenchymal transition, growth, migration, and resistance to apoptosis (75). However, in xenograft models, GC cells with silenced FPR1 grow more rapidly to form larger tumors in immune deficient mice. Mechanistically, tumors derived from GC cells with FPR1 knockdown contain vasculature with higher density. Therefore, suggesting FPR1 may participate in anti-angiogenic process in GC, depriving tumors of nutrients.

In contrast to observations with GC cells, FPR1 expressed by highly malignant human glioblastoma (GBM) cells (76) by responding to a ligand DAMP annexin (Anx) A1 released by necrotic tumor cells in the microenvironment, trans-activates EGFR and these receptors coordinate to promote GBM cell survival, invasion, and angiogenic factor production (76–80). The contribution of FPR1 to GBM progression was further verified by experiments in which siRNA targeting FPR1 greatly diminished the tumorigenicity of GBM cells in xenograft mouse models. In addition, FPR1 may participate in GBM tumor initiation, because CD133/Nestin positive glioma stem-like cells (GSCs) express FPR1 (81) and form more rapidly growing xenograft tumors and release increased angiogenic cytokines upon FPR1 activation. Moreover, GBM cells with necrosis release an FPR1 agonist Anx A1that actives the receptor on tumor cells to exacerbate the invasive behavior. These observations establish a paradigm of FPR1/Anx A1 axis as a critical component in the GBM microenvironment promoting tumor progression (82). Studies of human primary glioma specimens demonstrate the co-expression of FPR1 and Anx A1 in more highly progressive tumors indicating the clinical relevance of the receptor and a tumor-derived ligand (76, 82).

Human breast cancer cells also express aberrant FPR1 and 2, which are activated by Anx A1 to increase tumor cell growth (83). Similar to observations in human GBM, FPR1 is detected in human liver cancer cells to promote cell migration, invasion, proliferation and production of angiogenic factors. The tumorigenicity of human liver cancer cells in immuno-compromised mice was markedly diminished by FPR1 knockdown. Therefore, FPRs are utilized by various malignant tumors for accelerating their progression and are potential targets for therapeutic development. As supporting evidence, the chemotaxis inhibitory protein derived from *S. aureus* (CHIPS), as an FPR1inhibitor, improved the survival rate of mice implanted with human GBM (84).

## Regulation of FPRs

FPRs expressed by leukocytes and tumor cells are subjected to regulation by a variety of PAMPs and cytokines that control cell function in complex microenvironment milieu (32–34). This provides the opportunity to better understand the mechanistic basis of many diseases as well as to benefit the design of therapeutic strategies at different molecular levels.

### Regulation of FPR Expression by Inflammatory Stimulants and Cytokines

**Figure 2:**
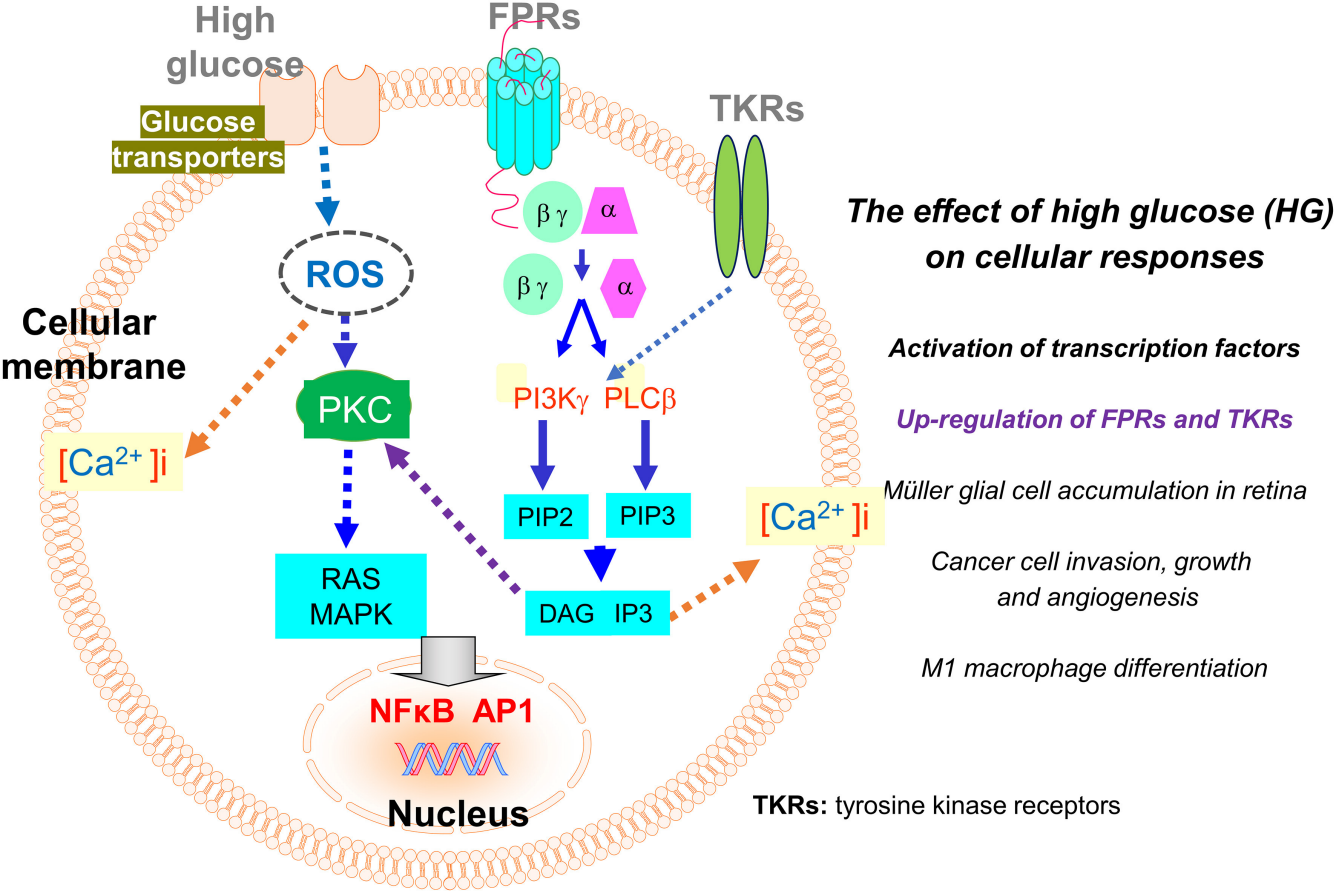
Regulation of the expression of FPRs and tyrosine kinase receptors (TKRs) by high glucose (HG). The expression of FPRs (Fprs) on inflammatory and tumor cells is up-regulated by HG through a ROS-PKC-MAPK and NFκB/AP1 pathway. The enhanced expression of Fpr2 and bFGFR on Müller glial cells promotes cell infiltration and expansion in the retina to exacerbate diabetic retinopathy. On human glioblastoma (GBM) cells, enhanced expression of FPR1 and EGFR induced by high glucose promotes cell chemotaxis, tumorigenesis, invasion, and production of angiogenic vascular endothelial growth factor (VEGF) to exacerbate the malignant phenotype.

The expression of FPRs on leukocytes and other cell types is regulated by pro- and anti-inflammatory signals. For instance, in murine brain microglial cells, several pro-inflammatory mediators such as LPS, TNFα, CD40/CD40 ligand, and other TLR agonists, up-regulate the expression of Fpr2, which mediates the chemotactic response of the cells to amyloid 1–42 (Aβ42), a pathogenic factor in Alzheimer's disease (AD). The increased expression of Fpr2 by microglial cells also promotes endocytosis and degradation of Aβ42 by the cells, a consequence thought to be beneficial for the host. However, long term exposure of microglial cells to high concentration of Aβ42 causes accumulation of the amyloid peptide in the cells to form the core for plaques, resulting in breakdown of the cells and inflammatory responses detrimental to neuronal cells (34). The ability of proinflammatory stimulants to enhance the expression of functional Fpr2 in microglial cells was attenuated by anti-inflammatory cytokines such as IL-4 (85) and TGFβ1 (86) through interference of the activation of key intracellular pathways such as MAPKs and NFκB. As consequence, the chemotaxis response to and phagocytosis of Aβ42 shown by microglial cells are diminished. However, whether the inhibitory effect of anti-inflammatory cytokines on Fpr2 induction by pro-inflammatory stimulants is beneficial remains unclear. Nevertheless, a logical view is that the fine-tuning of Fpr2 function in microglial cells should be important for amplifying host clearance of abnormally aggregated Aβ42 peptides and in the meantime, reducing neuronal damage caused by overt inflammatory responses [Figure 2; (34)].

### Regulation of FPR Expression by High Glucose

It is of great interest that FPRs expressed by immune and cancer cells are also subjected to regulation by glucose. Hyperglycemia (or high glucose, HG), as a hall marker of diabetes mellitus, arises when the host body is unable to maintain a normal serum level of glucose. Hyperglycemia induces over-production of reactive oxygen species (ROS) that causes oxidative stress disrupting intracellular metabolic cycles, signal transduction capacity, and cell-to-cell cross-talk associated with activation and translocation of transcription factors including NF-κB. These events result in the release of increased levels of inflammatory cytokines, chemokines, and prostaglandins (87). Inflammatory mediators perturb immune function that interferes with detection and clearance of pathogenic microorganisms and tissue debris. In addition, inflammation associated with hyperglycemia contributes to the more rapid growth of cancer (32, 88, 89). Recent studies revealed that FPRs expressed by Müller glial cells (MGCs) in the retina and human glioblastoma multiforme (GBM) cells are enhanced by HG, which imply the potential role of these GPCRs in the progression of diabetic retinal disease and further confirms their participation in the progression of human GBM (Figure 2).

#### Diabetic Retinopathy (DR)

DR is associated with diabetes that causes vison damage. The retinas of patients with diabetes show a number of pathological conditions related to inflammatory responses (90). Based on the severity of the pathology, DR is categorized into mild, moderate, severe non-proliferative stages, to advanced proliferative DR (PDR), which is characterized with the presence of expanding neo-vasculature and fibrovascular tissues extending from the retina to “invade” the vitreous. The ensuing increased force of traction causes vitreous hemorrhage, and retinal detachment, culminating in irreversible vision loss. These pathological processes suggest the involvement of hyperglycemia-associated inflammation as a foundation for DR (91).

In PDR, MGCs increase their motility and proliferation that participate in the formation of fibrovascular membrane. Recent studies reveal that HG enhances the expression of Fpr2 and FGFR1 (fibroblast growth factor receptor 1) by MGCs to promote cell chemotaxis and growth. Mechanistically, HG activates NF-κB and increases the phosphorylation of MAPKs downstream of Fpr2 and FGFR1 in MGCs, which also increase the release of VEGF. In diabetic mice, MGCs in the retina express higher levels of Fpr2 and its human analog FPR2 is revealed in MGCs in the fibrovascular membrane of the abnormal retina in PDR patients. In support of pathological relevance of FPR2 (Fpr2) and the ligand pair in PDR, an endogenous Fpr2 agonist CRAMP is expressed in mouse MGCs and retinal tissues, which is further increased by HG in cultured MGCs (92). Since FPRs are reported to promote inflammation and angiogenesis in PDR vitreous, it is plausible to target these receptors to ameliorate the severity of PDR (93).

#### Human GBM

As discussed earlier, GBM is associated with very poor survival in patients despite multidisciplinary therapies (94). Although aging, male gender and white ethnicity are putative risk factors for GBM (87), HG is found to accompany increased malignancy of high grade gliomas and the rate of recurrence (88).

Recent studies demonstrate that *in vitro*, HG favors more rapid growth of a human GBM cell line (95). As a mechanistical basis, HG treatment of GBM cells results in increased the expression of functional FPR1 and EGFR by eliciting enhanced activation MAPKs and NFκB. FPR1 and EGFR by their respective agonists to mediate tumor cell migration, growth and formation of cell colonies. HG also increases the invasion and VEGF production by GBM cells, which are exacerbated by FPR1 and EGFR activation by their ligands. The ability of HG to promote tumor progression was supported by increased growth rate of tumors formed by GBM cells in diabetic nude mice (95). These observations clearly show the potential of HG to promote GBM progression by enhancing the function of FPR1 and EGFR. Nevertheless, although diabetes has been shown to be associated with more rapid progression of GBM and higher patient mortality, the connection with the levels of FPR1 and EGFR in tumor remains to be established in the clinic samples.

### Involvement of Fpr2 in Promoting Mouse Insulin Resistance and Obesity

Although FPRs are subject to regulation by glucose levels, Fprs was recently shown to exacerbate host responses to glucose, support insulin resistance, and obese complications in mice on high fat diet (HFD). Obesity and accompanying inflammation are critical for the development of insulin resistance. As discussed earlier, Fpr2 promotes the M1 polarization of TAMs and limit tumor growth a mouse LLC models (67). However, this property of Fpr2 was exploited by a metabolic disease for recruitment and M1 polarization of macrophages in white adipose in mice with HFD-induced obesity. Mice with systemic deficiency of Fpr2 demonstrate reduced severity of HFD-induced obesity, insulin resistance, hyperglycemia, hyperlipidemia, and hepatic steatosis (97). These mice also contained reduced fat mass in the body and inflammation with reduction in macrophage accumulation and M1 polarization in adipose tissues. Further studies indicate Fpr2 expressed by myeloid cells as a major contributor of HFD-induced mouse obese syndrome. Mechanistically, adipose tissue-derived Fpr2 agonists may induce macrophage recruitment. Therefore, the capacity of Fpr2 to promote M1 polarization appears to be a double-edged sword which favors host anti-cancer defense but exacerbates the progression of metabolic diseases.

## Perspectives

FPRs belong to classic chemoattractant GPCRs expressed by a variety of cell types including immune cells, cells of the non-hematopoietic origin, and cancer cells, These GPCRs possess one of the most diverse arrays of ligands of both pathogen (PAMPS) and host (DAMP) sources. Accumulating evidence places FPRs in unique positions in mediating leukocyte trafficking, colon mucosal homeostasis, and in host resistance to tumorigenesis. The functions of FPR family members are dependent largely on cell types, ligands, tissue microenvironment and more importantly, disease conditions. Many novel developments obtained in the past few years substantiated the understanding of the involvement of FPR in pathophysiology. Nevertheless, further exploration of the participation of FPRs in a greater number of diseases has become more feasible with the generation of mice with mutated genes coding for Fprs and ligands. Therefore, studies of FPR (Fpr) regulation, signaling, structure/function relationship in various cell types, and more importantly, in diseases, especially those of human, should aid in the discovery of novel medicines.

## Author Contributions

WL collected the data and drafted the manuscript. KC, WG, TY, YL, YW, and JW collected the data. JW devised the main concept of manuscript and contributed to the revision of draft. All read and approved the submitted version.

### Conflict of Interest

WG was employed by the company Leidos Biomedical Research, Inc. The remaining authors declare that the research was conducted in the absence of any commercial or financial relationships that could be construed as a potential conflict of interest.
